# PADI4 promotes epithelial-mesenchymal transition(EMT) in gastric cancer via the upregulation of interleukin 8

**DOI:** 10.1186/s12876-022-02097-0

**Published:** 2022-01-19

**Authors:** Xiao-tian Chang, Hui Wu, Hui-lin Li, Hong-lei Li, Ya-bing Zheng

**Affiliations:** 1 Department of Oncology, The First Affiliated Hospital of Shandong First Medical University & Shandong Provincial Qianfoshan Hospital, Shandong Key Laboratory of Rheumatic Disease and Translational Medicine, Shandong Lung Cancer Institute, Jinan, 250014 Shandong China; 2 Medical Research Center of The Affiliated Hospital of Qingdao University, and Qingdao Engineering Center of Major Disease Markers, Qingdao, 266000 Shandong China; 3 Shandong Provincial Qianfoshan Hospital, Jinan, 250014 Shandong China

**Keywords:** Gastric cancer, Metastasis, EMT, Peptidyl arginine deiminase IV, Signaling pathway

## Abstract

**Background:**

Gastric cancer (GC) is one of the deadliest tumours due to its ability to metastasize. The Epithelial–to-mesenchymal transition plays a crucial role in promoting the GC metastasis, which increases the migration and metastasis of tumour cells. Peptidyl arginine deiminase IV (PADI4) is a susceptibility gene for gastric carcinoma. The aim of this study was to evaluate the functional roles of PADI4 in gastric cancer.

**Methods:**

The expression of PADI4 was examined by qRT-PCR, western blot and immunohistochemistry. In addition, the functional roles of PADI4 were explored by over-expression PADI4 plasmids in gastric cancer cells.

**Results:**

We found that the expression of PADI4 was up-regulated in GC. PADI4 overexpression in GC cells increased the proliferation, migration, metastasis, clone forming ability, and tumorigenic ability, but reduced the apoptosis ability. The Multi-Analyte ELISArray Kit results showed that interleukin 8 (IL-8) is upregulated in PADI4-overexpressing gastric cells. Using short interfering RNA (siRNA) to silence the expression of IL-8, we demonstrated that IL-8 silencing significantly inhibited the increased migratory capacity in PADI4-overexpressing GC cells.

**Conclusions:**

Our data suggest that PADI4 accelerate metastasis by promoting IL-8 expression in gastric cancer cells, indicating that it is a new PADI4/IL-8 signalling pathway in metastatic GC.

## Background

Gastric cancer (GC) is a common cancer in the world [1]. This disease is mostly diagnosed at the late stage and accompanied by metastasis [2]. The current treatment for metastatic GC is limited and ineffective. The median overall survival of metastatic GC ranges from 9 to 13 months despite treatment with systemic chemotherapy [3]. Metastasis is the main cause of death in GC. Therefore, it is essential to understand the pathogenesis of GC metastasis, which will help us to find more approaches for the treatment of metastatic GC. Recent studies have found that epithelial-mesenchymal transition(EMT) play an important role in provide cancer cells with mobility [4]. The EMT is the process by lose their epithelial characteristics and acquire migratory and invasive properties characteristic of cancer cells [5]. This process is accompanied by decreased expression of epithelial markers(E-cadherin) and increased expression of mesenchymal markers (neural cadherin and vimentin). A group of paracrine factors, including Snail family and zinc-finger transcription factors, promote the expression of EMT at the translational and posttranslational levels [6]. Therefore, it is very important to explore the mechanism of EMT in the pathogenesis of gastric cancer.

Peptidyl arginine deiminase IV (PADI4) is a calcium-dependent enzyme, and its role is to convert arginine to citrulline [7]. Citrullination mediated by peptidyl arginine deaminase plays a major role in the functional and structural stability of proteins and then affects the physiological and pathological processes of the body [8]. Increasing evidence suggests that PADI4 is involved in tumour progression [9]. We and others have found that PADI4 is overexpressed in many cancers, including ovarian cancer, colorectal cancer, lung cancer, cervical squamous cell carcinoma and thyroid carcinoma [10]. The pathological classification of oesophageal squamous cell carcinoma was positively correlated with the expression level of PADI4 [7, 11]. Moreover, it has been reported that PADI4 plays a crucial function in tumour cell proliferation, apoptosis and EMT [12]. We have reported that PADI4 can increase ovarian cancer cell line A280 proliferation, invasion and migration via the p53 signalling pathway [13]. In addition, we have reported that PADI4 is a susceptibility gene for GC. PADI4 upregulates the expression levels of KRT14, CXCR2 and TNF-α, which are related to cell proliferation, cell migration, tumour angiogenesis, and tumour immune microenvironment [14]. However, the role of PADI4 in the development of GC, especially in gastric metastases, is poorly understood.

Interleukin-8 (IL-8, also called CXCL8) is a cytokine of the CXC chemokine family that is believed to be related to cancer development and chronic inflammation [15]. IL-8 can activate cell surface G protein-coupled receptors (CXCR1 and CXCR2) in various signalling pathways [16]. As a very important autocrine regulator factor in the tumour microenvironment, IL-8 and/or its receptors play a crucial role in regulating tumour growth and metastasis, especially in human gastric carcinoma cells [17, 18]. Overexpression of IL-8 promotes cellular proliferation, metastasis, invasion, adhesion in GC cells [16]. Fu et al. found that IL-8 is involved in the EMT process [19].

In this study, we found that PADI4 can promoted the proliferation, migration and and inhibited the apoptosis of GC cells. Further studies showed that these functions are achieved by controlling the expression of IL-8. Those results of this study provide a new mechanism for the role of PADI4 in GC metastasis and provide a reasonable explanation for the correlation between the high expression of PADI4 and clinical stage of gastric cancer, suggesting that PADI4 may become a potential therapeutic target for advanced gastric cancer.

## Methods

### Tissue collection

Tumour tissues(n = 7) and normal adjacent mucosal tissues(n = 7) were collected from 30 to 70 years old from April 2017 to June 2019 at Shandong Province Qianfoshan Hospital. The distance between the tumour tissues and normal adjacent mucosal tissues was 5 cm. The inclusion and exclusion criteria for the patients were based on the method of Wang B et al. [20].

Written informed consent was obtained from each participating individual, our study was approved by the Ethics Committee of Shandong Provincial Qianfoshan Hospital, all methods were performed in accordance with the International Conference on Harmonization Guideline for Good Clinical Practice (E6) and the 2013 Declaration of Helsinki. Tumour diagnosis performed according to the World Health Organization(WHO) classification system.

### Western blotting

Total protein was lysed from GC tissues or cells by using RIPA lysis solution (Solarbio, China). Thirty micrograms of total protein extract was separated by 10% SDS-PAGE and then transferred to a PVDF membrane, the PVDF membrane was then incubated with 5% skim milk for 1 h. Following being incubated with primary antibodies (1:1,000) overnight at 4 °C. Then washed three times, the membrane was incubated with HRP-conjugated secondary antibody (1:5,000) for 1 h. The signal was detected using enhanced chemiluminescence (ECL) with an Alpha FluorChem E (Cell Biosciences, USA). The primary antibodies targeting PADI4 (ab128086; Abcam); GAPDH (ab181603; Abcam); CXCL8 (DF6998; Affinity); vimentin (cat. no. 5741; Cell Signaling Technology) and E-cadherin (cat. no. 3195; Cell Signaling Technology) were used in this study.

### Real-time PCR

Total RNA was isolated from GC tissues or cells(Invitrogen, USA). One microgram of total RNA was reverse transcribed into first-strand cDNA (Vazyme, China), which was then used as the PCR template. Real-time PCR was performed using Bio-Rad CFX96 detection system. The primers were listed in Table 1. Data were analysed by the formula: R = 2^−[ΔCt sample−ΔCt control]^.

**Table 1:** List of primers used for quantitative PCR

Primer	Sequence(5'–3')
PADI4-Forward	5’-GGGGTGGTCGTGGATATTGC-3’
PADI4-Reverse	5’-CCCGGTGAGGTAGAGTAGAGC-3’
GAPDH-Forward	5’-CAGAACATCATCCCTGCCTCTAC-3’
GAPDH-Reverse	5’-TTGAAGTCAGAGGAGACCACCTG-3’

### Immunohistochemistry

GC tissue array was obtained commercially from Alenabio (cat. no. ST242). The array slides contained 20 gastric cancer and 4 normal tissue samples. The tissue sections were deparaffinized and rehydrated by standard procedures. After antigen retrieval and incubation with an endogenous peroxidase inhibitor (Maixin, China), the array slides were incubated with the anti-PADI4 antibody overnight. Then, the tissue array were incubated with secondary antibody (1:500; cat. no. A0216; Beyotime) for 30 min. Immunocomplexes were detected using diaminobenzidine (DAB) for 3 min and washed in water for 10 min. The slides were then stained with haematoxylin. The result of the IHC for PADI4 was judged based on Liu H et al. method [21].

### Cell culture

The human GC cell lines(SGC-7901, MGC80-3)were obtained from GeneChem. SGC-7901 is a moderately differentiated cell originating from lymph node metastases with gastric adenocarcinoma. MGC80-3 is a poorly differentiated cell with gastric mucoid adenocarcinoma. SGC-7901 and MGC80-3 cells were cultured in DMEM (Gibco) containing 10% foetal bovine serum (Gibco). Cells were maintained in a humidified atmosphere containing 5% CO_2_ at 37 °C and passaged once every two days.

### Plasmid construction and transfection

The pcDNA 3.1(+)-RFP containing the full sequence of PADI4 was applied to overexpress PADI4. The pcDNA 3.1(+)-RFP or pcDNA 3.1(+)-PADI4-RFP plasmid were transfected into GC cells using PolyJet™ transfection reagent (SignaGen,USA) in accordance with the manufacturer’s protocol. Cells transfected with pcDNA 3.1(+)-RFP were used as negative controls. The transfected cells were then harvested for 48 h for further experiments.

### Wound-healing assay

After transfection, cells were plated into 6-well plates. After the cells reached 100% confluence, a scratch was made by a pipette tip, and a photo was taken at 0 and 24 h. Wound healing was imaged under the microscope.

### Transwell migration assay

GC cells after transfection in six plates for 24 h. Then, the upper chambers (3412; Corning) were added with serum-free culture medium containing 1*10^5^ transfected cells. After cultured for 24 h, cells were immersed in methanol and stained with 600 µl 1% crystal violet. The cells were counted with the microscope.

### Cell proliferation

Cell Counting Kit 8 (CCK-8) (Dojindo, Japan) assays were performed to assess cell proliferation. Cultured cells were cultured in 96-well plates with the initial density of 5,000 cells/well. The cells were transfected with pcDNA 3.1(+)-RFP or pcDNA 3.1(+)-PADI4-RFP as described above. At 24 h, 48 h after transfection, CCK-8 was added into the cell culture medium. The absorbance was determined at 450 nm using SpectraMax 190 (Molecular Device).

### Colony formation assay

One thousand GC cells were transfected with pcDNA 3.1(+)-RFP or pcDNA 3.1(+)-PADI4-RFP as described above after being seeded in 60 mm cell culture dishes. Then, the cells were incubated for two weeks until colonies appeared. After being fixed with methanol and stained using 1% crystal violet for 30 min, the colonies (each colony with at least 50 cells) were counted.

### Cell apoptosis assay

GC cells after transfection in six plates for 48 h, and then washed and resuspended. The cell suspensions were transferred into tubes, and 1.5 μl of crystal violet (eBioscience; USA) was added for 30 min. Then, 5 μl of fluorescein isothiocyanate (FITC) (eBioscience; USA) were added to the tube for 15 min in the dark.

### Xenograft model

Male nude mice (5 weeks; 17–18 g) were housed in a pathogen-free animal facility. All procedures were approved by the Committee for Animal Research at Shandong Provincial Qianfoshan Hospital, and were performed according to the guidelines for animal care and use. The pHBLV-CMV-6*His-EF1-mCherry-puro lentiviral vector containing full coding sequence of PADI4 was applied to overexpress PADI4 (Hanbio, China). Recombinant pHBLV-CMV-PADI4-6*His-EF1-mCherry-puro viruses were transfected into MGC80-3 cells (1*10^7^/ml) injected into the left flank of each mouse to generate tumour-bearing BALB/c nude mice; pHBLV-CMV-6*His-EF1-mCherry-puro viruses were transfected into MGC80-3 cells as the control group and injected into the right flank of each mouse (n = 8).Tumours were dissected 40 days after cell injection, and the size and weight of the tumours were measured by routine methods.

### Multi-analyte ELISArray and ELISA analyses

GC cells after transfection in six plates for 48 h, the Multi-Analyte ELISArray Kit(cat. no. MEM-004A) (Qiagen, USA) were used to screen cytokines performed the manufacturer’s instructions. This kit can measure the levels of IL-1α, IL-1β, IL-2, IL-4, IL-6, IL-8, IL-10, IL-12, IL-17A, interferon-γ(IFN-γ), tumour necrosis factor-α(TNF-α) and granulocyte–macrophage colony-stimulating factor (GM-CSF). The absorbance levels of these cytokines were measured on a plate reader (SpectraMax 190, Molecular Device) at 450 nm. We tested the expression level of IL-8 by ELISA analyses.

### siRNA transfection

Anti-IL-8 siRNA (target mRNA sequence: 5’-GCAGAAGGUUGUACAGAUATT-3’), anti-PADI4 siRNA(target mRNA sequence: 5’-CAGCGTAGTCTTGGGTCCCAA-3’) and nonsilencing siRNA (sequence 5’-UUCUUCGAACGUGUCACGUTT-3’) were synthesized by GenePharma Co. Ltd. The cultured GC cells were transfected with the anti-IL-8 siRNA or anti-PADI4 siRNA using PepMute™ siRNA transfection reagent(SignaGen, USA) according to the manufacturer’s instructions.

### Statistical analysis

Data are presented for experiments performed in triplicate and are expressed as mean ± standard deviation(SD). SPSS 19.0 software was used for the experimental data analysis. Statistical analysis was performed with Student’s t-test between two groups or one-way ANOVA for multiple groups. *p < 0.05, **P < 0.01, ***P < 0.001.

#### Ethics approval

This study was approved by the Ethics Committee of Shandong Provincial Qianfoshan Hospital with the mumber of 2017(S090). All methods were performed in accordance with the relevant guidelines and regulations.

## Results

### PADI4 is overexpressed in GC tissues

**Fig. 1:**
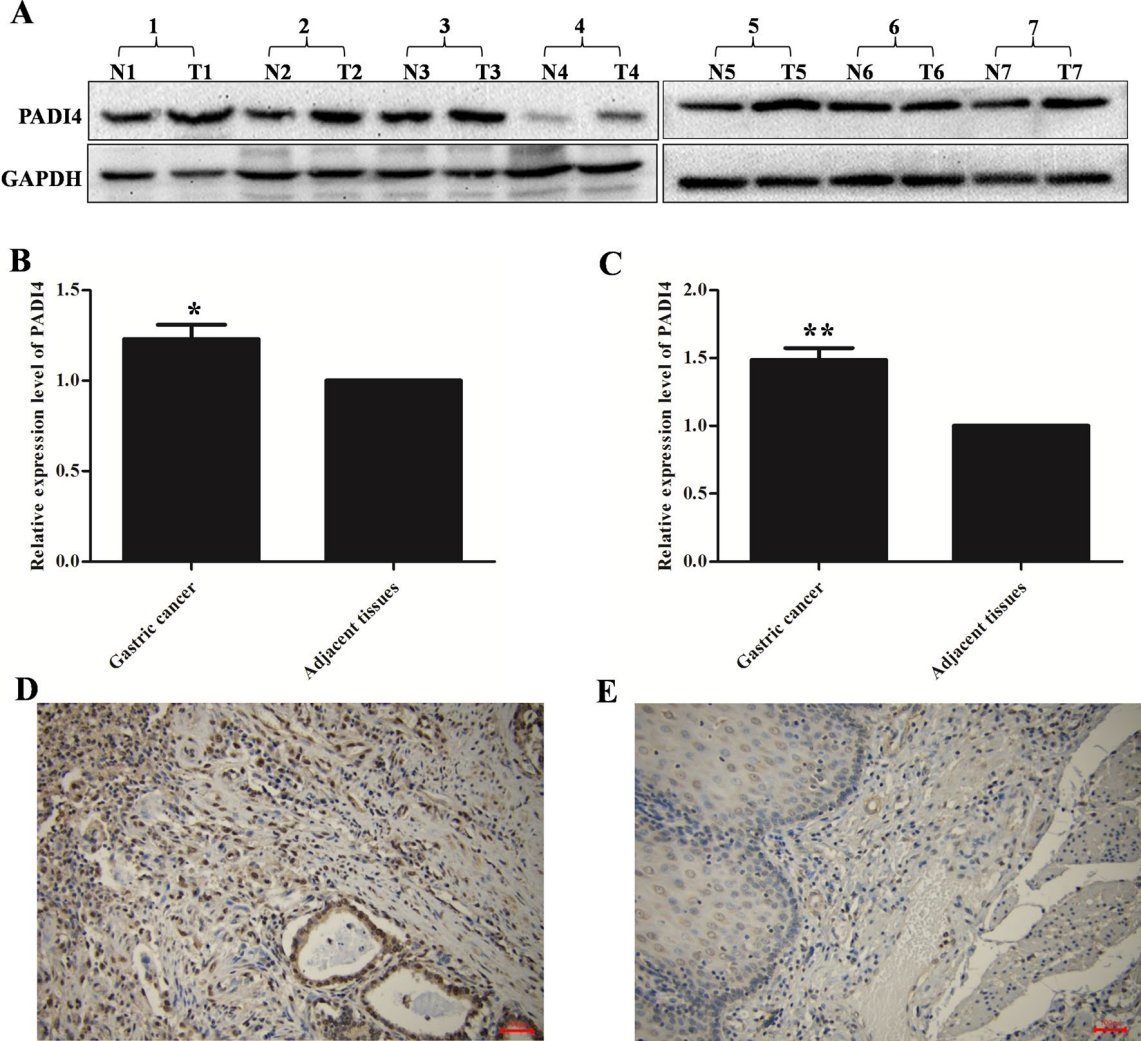
PADI4 expression in GC tissues. PADI4 expression levels in GC tissue samples and corresponding adjacent tissue samples were determined by western blot (**A**, **B**) and qRT-PCR (**C**). PADI4 expression was detected using immunohistochemistry in different GC tissues (**D**) and normal gastric tissues (**E**) T: tumour tissues; N: corresponding adjacent tissues. Original magnification: 200×

Analysis of tissues revealed that PADI4 protein expression levels in GC tissue tumour samples were increased compared with those in the corresponding adjacent tissues (*p* = 0.03; Fig. 1A, B). Similarly, PADI4 mRNA expression measured by real-time PCR was also significantly increased in GC tumour samples compared with paired adjacent normal gastric tissue samples (*p* = 0.002; Fig. 1C).

We detected the expression of PADI4 in GC tissues by immunohistochemistry. The results showed that PADI4 was positive in GC tissues, such as nasointestinal tube and mucinous cystadenocarcinoma of GC patients but was limited in normal gastric tissues (Fig. 1D, E).

### Effect of PADI4 on GC cell migration, proliferation, colony formation and apoptosis

**Fig. 2:**
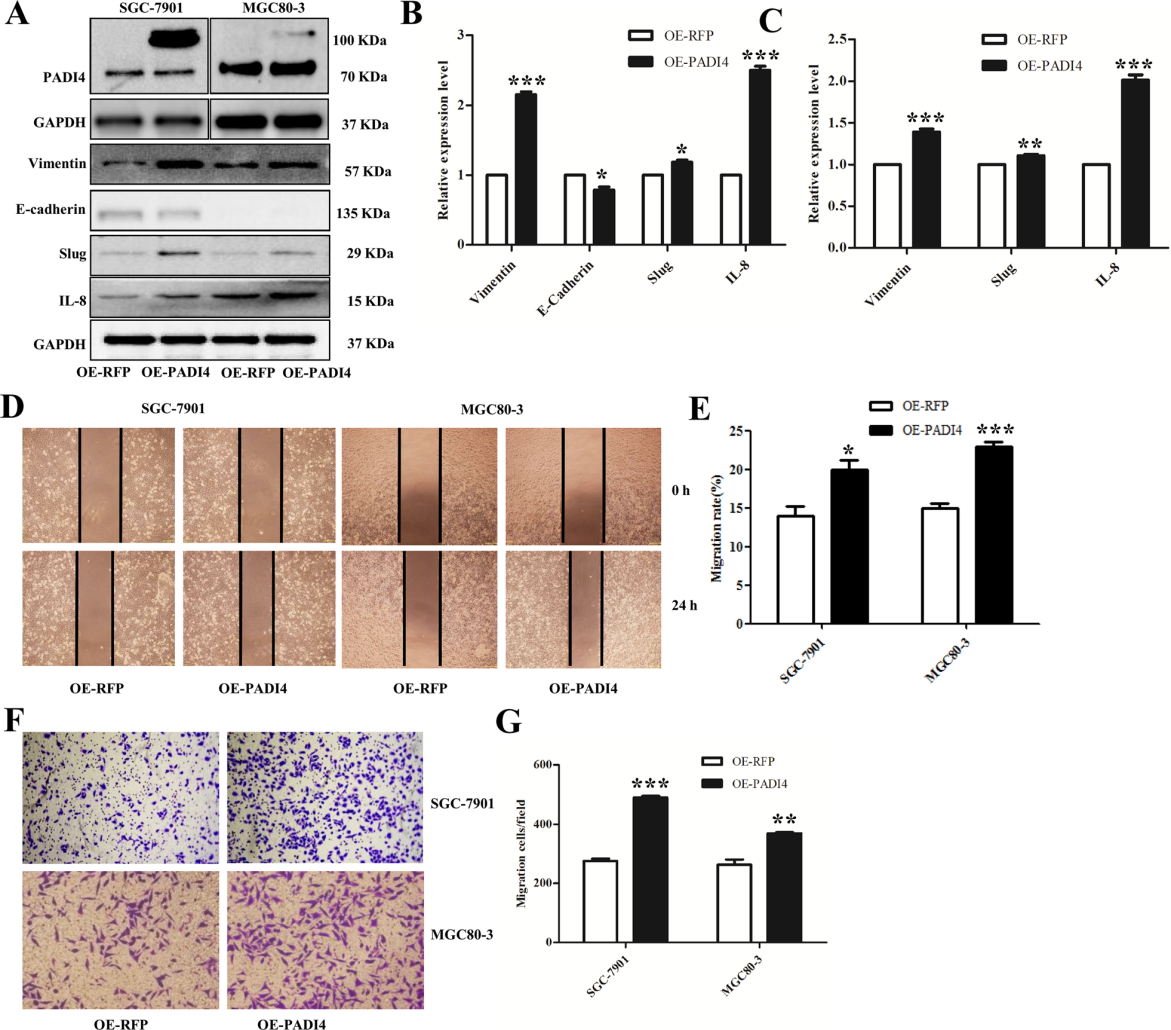
Role of PADI4 overexpression on the migration of GC cells. SGC-7901 and MGC80-3 cells were transfected with plasmid. The protein expression of PADI4, IL-8, E-cadherin, vimentin and Slug was determined by western blot (**A**–**C**). Cell migration was determined by wound healing assay (**D**, **E**) (Original magnification: 100×) and Transwell assay (**F**, **G**) (Original magnification: 400×). All results were obtained from three independent experiments

GC cells were transfected with recombinant pcDNA3.1-RFP-PADI4 plasmid or pcDNA3.1-RFP plasmid. Western blot results found the increased expression of PADI4 (Fig. 2A). Therefore, the pcDNA3.1-RFP-PADI4 plasmid was used for further studies.

The role of PADI4 expression in the cell migration ability was investigated by wound healing assay and Transwell migration assay. The wound healing assay showed that the PADI4-transfected GC cells had a significantly higher migration rate (SGC-7901, *P* = 0.021; MGC80-3, *P* = 0.006) (Fig. 2D, E). In addition, Transwell migration assay exhibited that the PADI4-transfected SGC-7901 and MGC80-3 cells had 1.78- and 1.4-fold higher migration than that observed in the empty vector cells(SGC-7901, *P* = 2.7*10^–5^; MGC80-3, *P* = 0.004) (Fig. 2F, G). Moreover, PADI4 overexpression decreased expression of E-cadherin and increased expression levels of Slug and vimentin (*P* < 0.05; Fig. 2A–C). Hence, these results demonstrated that PADI4 can promote cell migration in vitro.

**Fig. 3:**
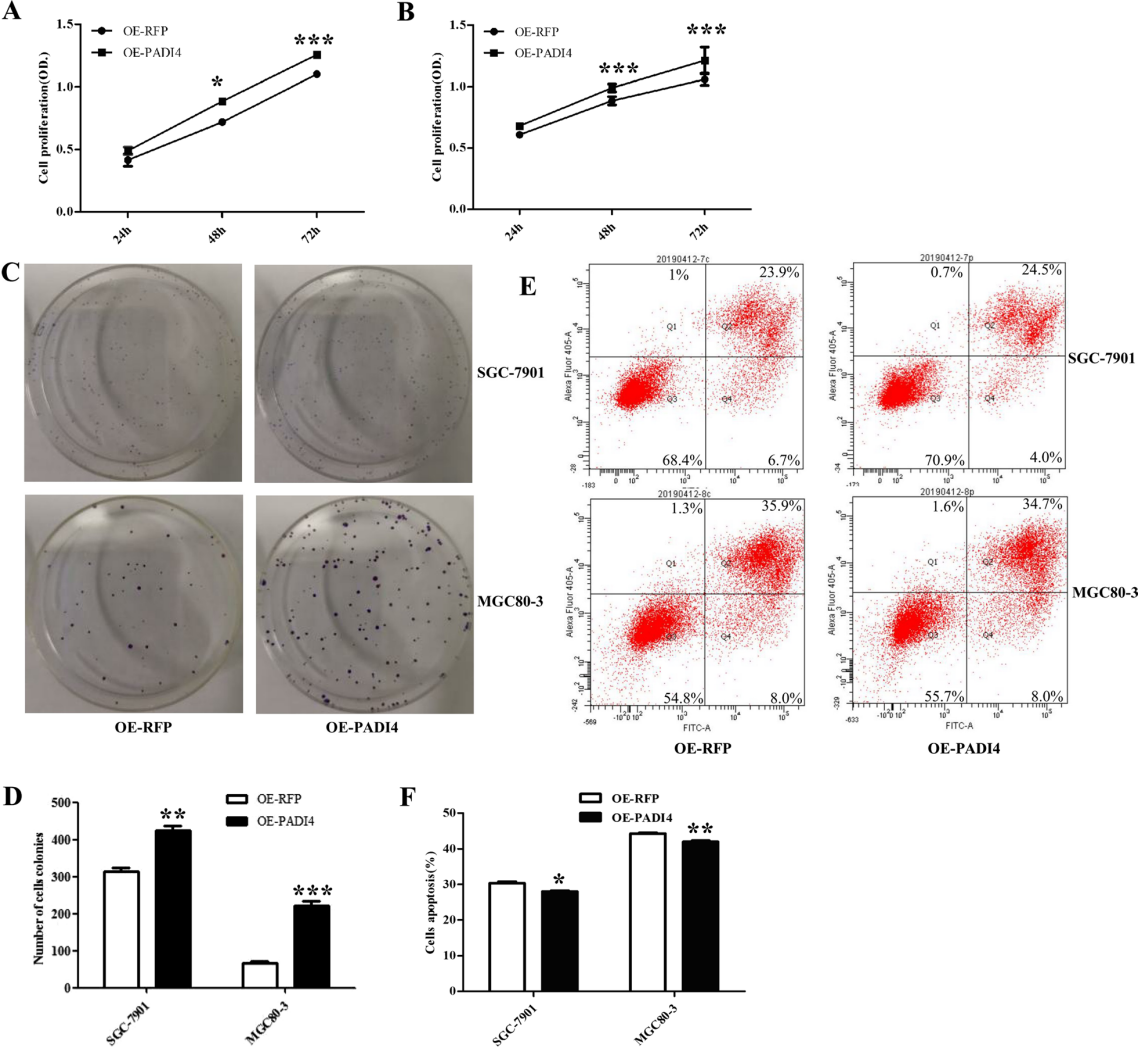
Effect of PADI4 overexpression on the proliferation and apoptosis of GC cell lines. Cell proliferation was determined by CCK-8 in SGC-7901 (**A**) and MGC80-3 (**B**) cells. The cell proliferation was determined by colony formation assays (**C**, **D**). The cell apoptosis ability was determined by flow cytometry (**E**, **F**)

The proliferation of GC cells was measured using CCK-8 and colony formation assays. We found that overexpression of PADI4 significantly increased the proliferation of GC cells at 24 h, 48 h and 72 h, indicating that PADI4 expression significantly increased the proliferation of GC cells (*P* < 0.05; Fig. 3A, B). Colony formation assays demonstrated that overexpression of PADI4 increased the number of colonies in GC cells (SGC-7901, *P* = 0.002; MGC80-3, *P* = 3.6*10^–4^) (Fig. 3C, D). These results indicate that PADI4 contributes GC cell proliferation and colony formation ability in vitro.

Flow cytometric analysis was used to measure the effects of PADI4 on GC cells apoptosis ability. The proportion of apoptosis was significantly decreased in GC cells transfected with pcDNA3.1-RFP-PADI4 plasmid compared with the cells treated with pcDNA3.1-RFP plasmid(SGC-7901, *P* = 0.014; MGC80-3, *P* = 0.006) (Fig. 3E, F).

### PADI4 promotes the growth of GC tumors in vivo

**Fig. 4:**
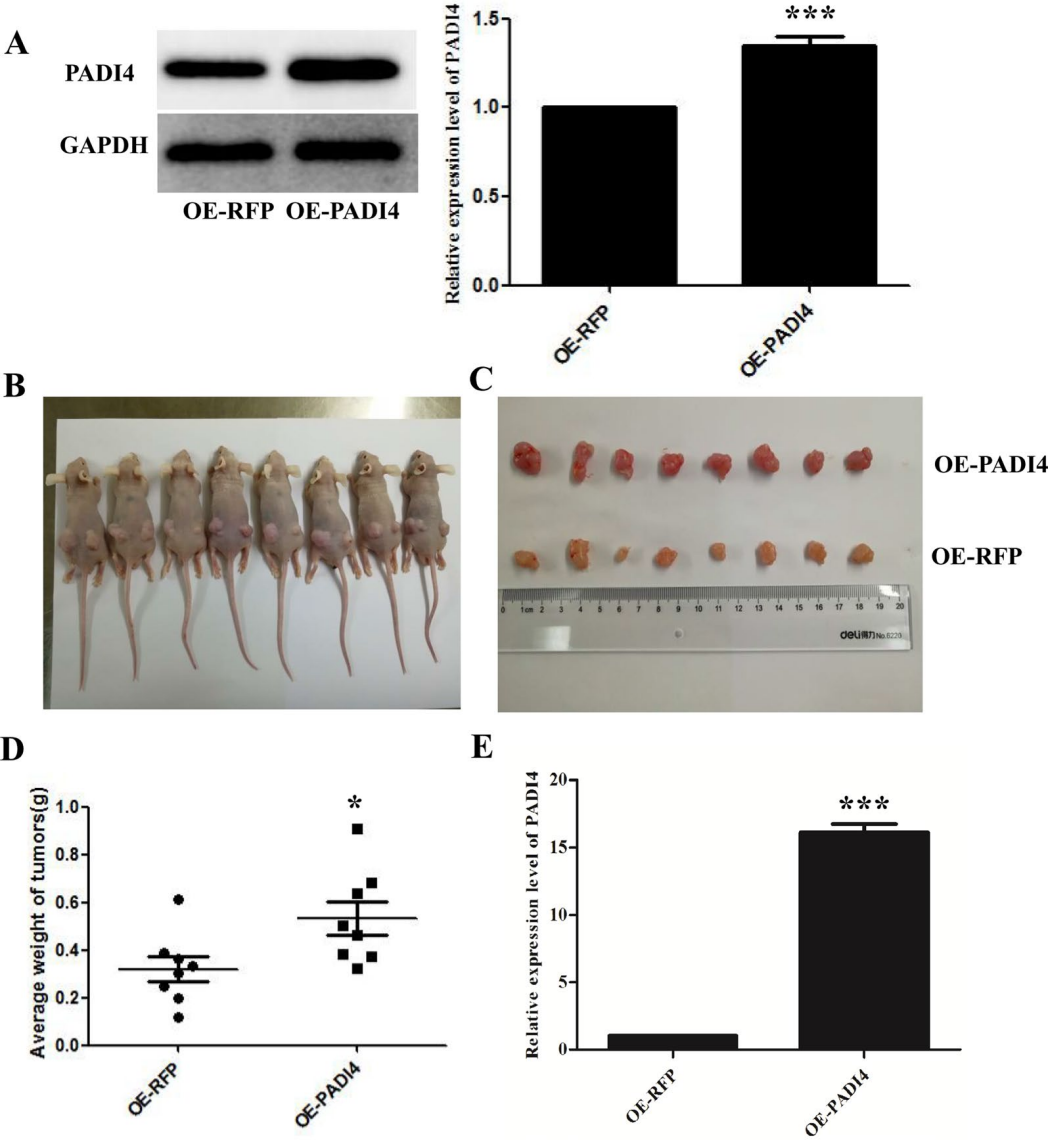
PADI4 promotes the tumorigenesis of GC cells in vivo. MGC80-3 cells were transfected with lentivirus, and the expression levels of PADI4 were determined by western blot analysis (**A**). The upper group of tumours (PADI4-overexpressing) and the lower group of tumours (control) (**B**, **C**). Tumour weights were measured and statistically analysed (**D**). The expression level of PADI4 in the tumour tissues was measured by qRT-PCR (**E**)

To confirm the role of PADI4 in GC in vivo conditions, we made a xenograft tumor model by subcutaneously injecting MGC80-3 cells stably over-expressing PADI4, the results showed that the average tumour weight in mice injected with PADI4-overexpressing virus transfected MGC80-3 cells was 534.7 mg, while the tumour weight of the control group was 320.7 mg (*P* = 0.012; Fig. 4A–D). These results demonstrate that increasing the expression of PADI4 can significantly accelerate cell activity and tumour growth in vivo. Additionally, our real-time PCR showed that tumours in mice injected with PADI4-overexpressing MGC80-3 cells exhibited strong PADI4 expression(P = 4.4*10^–8^; Fig. 4E). These results indicate that PADI4 can promote GC tumour growth in vivo.

### PADI4 regulates IL-8 expression

**Fig. 5:**
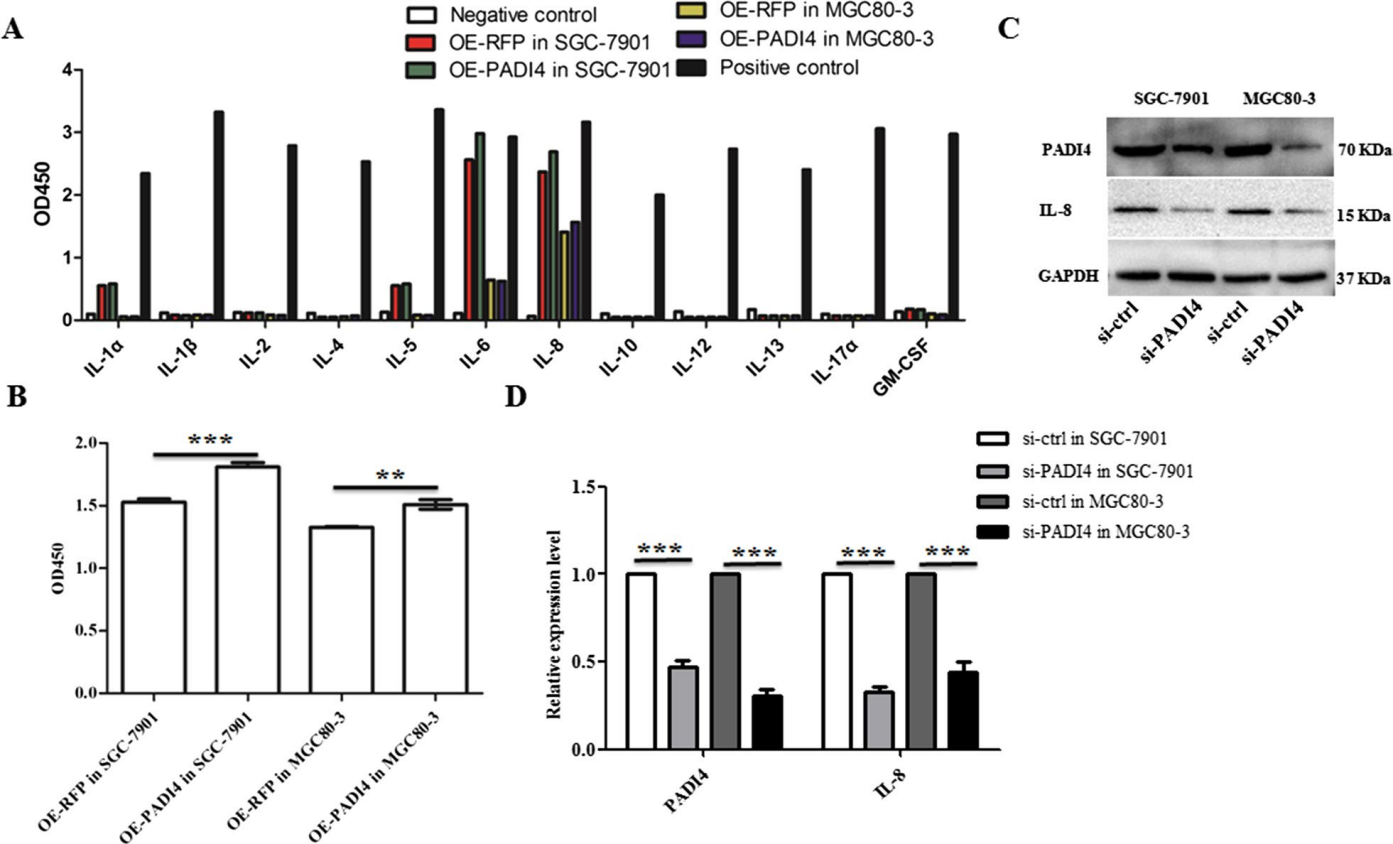
PADI4 regulates IL-8 expression. Cytokine release was measured by the human cytokine Multi-AnalyteELISA Array Kit in the supernatants of PADI4-transfected GC cells (**A**). The release of IL-8 protein in the cell supernatant was determined by ELISA (**B**). The protein expression of PADI4 and IL-8 was determined by western blot in si-PADI4 GC cells (**C**, **D**)

To better understand the mechanism of PADI4 involved in the progression of chronic inflammation in GC. We used a Multi-Analyte ELISArray Kit to screen cytokines in GC cell culture medium, and the results showed that PADI4-transfected GC cells produced a higher level of IL-1α, IL-6 and IL-8 compared with empty vector cells, but undetectable levels of release of cytokines IL-1β, IL-2, IL-4, IL-5, IL-10, IL-12, IL-13, IL-17A and GM-CSF in GC cell culture supernatants (Fig. 5A). To verify the above results, we performed ELISA and western blotting experiments, and the results showed that the GC cells transfected with the pcDNA3.1-PADI4-RFP plasmid produced a higher level of IL-8 than empty vector cells (*P* < 0.05; Fig. 2A and 5B). Next, siRNA against PADI4 was transfected to GC cells to reduce PADI4 expression. We also found that the expression of IL-8 protein in PADI4 knockdown GC cells was downregulated (*P* < 0.05; Fig. 5C, D). These data suggest that PADI4 regulates the expression of IL-8 protein.

### IL-8 knock-down reversed the effects of PADI4 on GC cells EMT and migration

We all know that IL-8 plays a vital role during the process of EMT [16], and we have found that PADI4 can regulate the expression of IL-8 and participate in the EMT process of gastric cancer (Figs. 2 and 5). This evidence prompted us to hypothesize that PADI4 regulates EMT function possibly by regulating the expression of IL-8 in GC. Thus, siRNAs targeting IL-8 were used in PADI4-overexpressing GC cells.

**Fig. 6:**
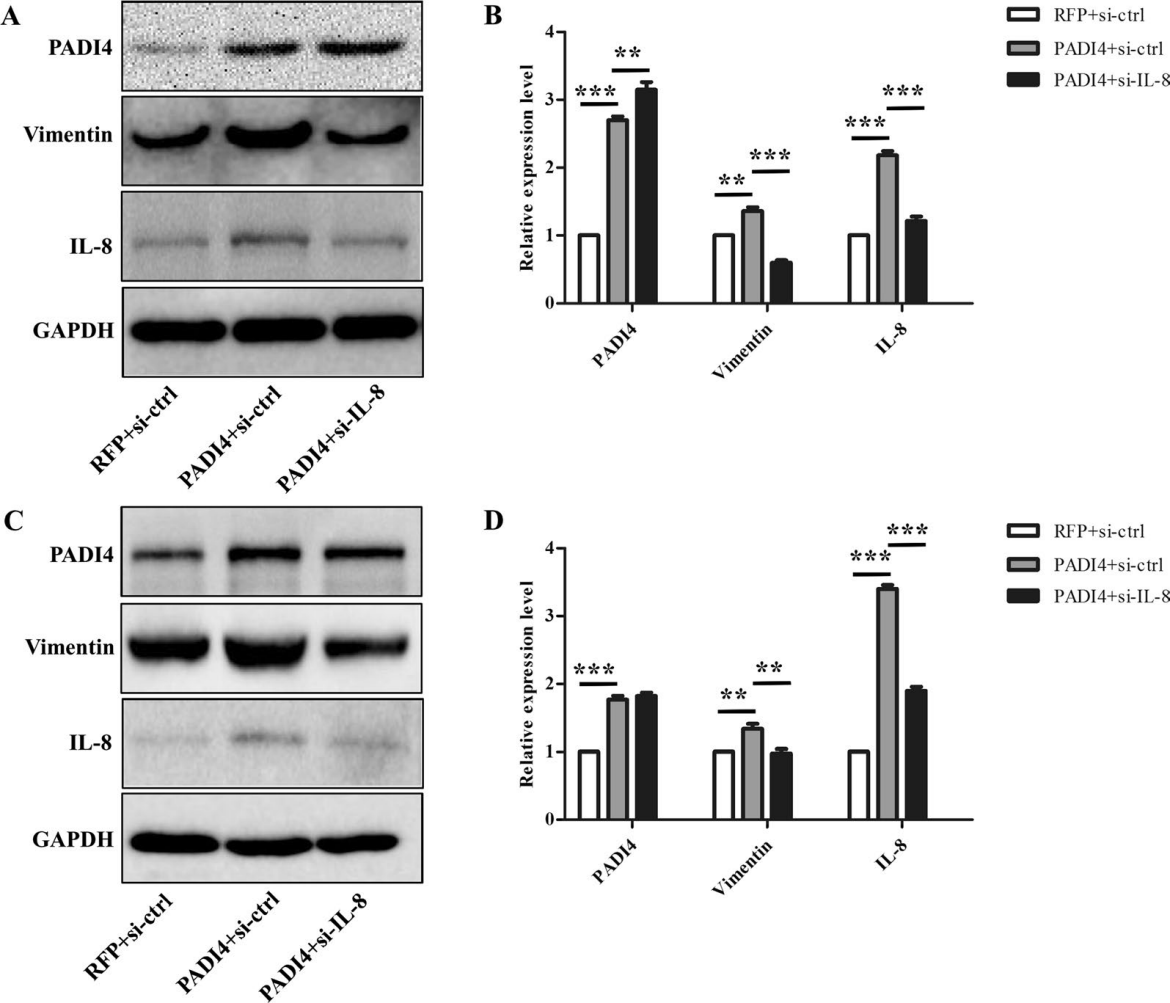
IL-8 blocks the effects of PADI4 on EMT. A. The protein expression of PADI4, IL-8 and vimentin was determined by western blot in MGC-803 cells (**A**, **B**). The protein expression of PADI4, IL-8 and vimentin in SGC-7901 cells (**C**, **D**)

**Fig. 7:**
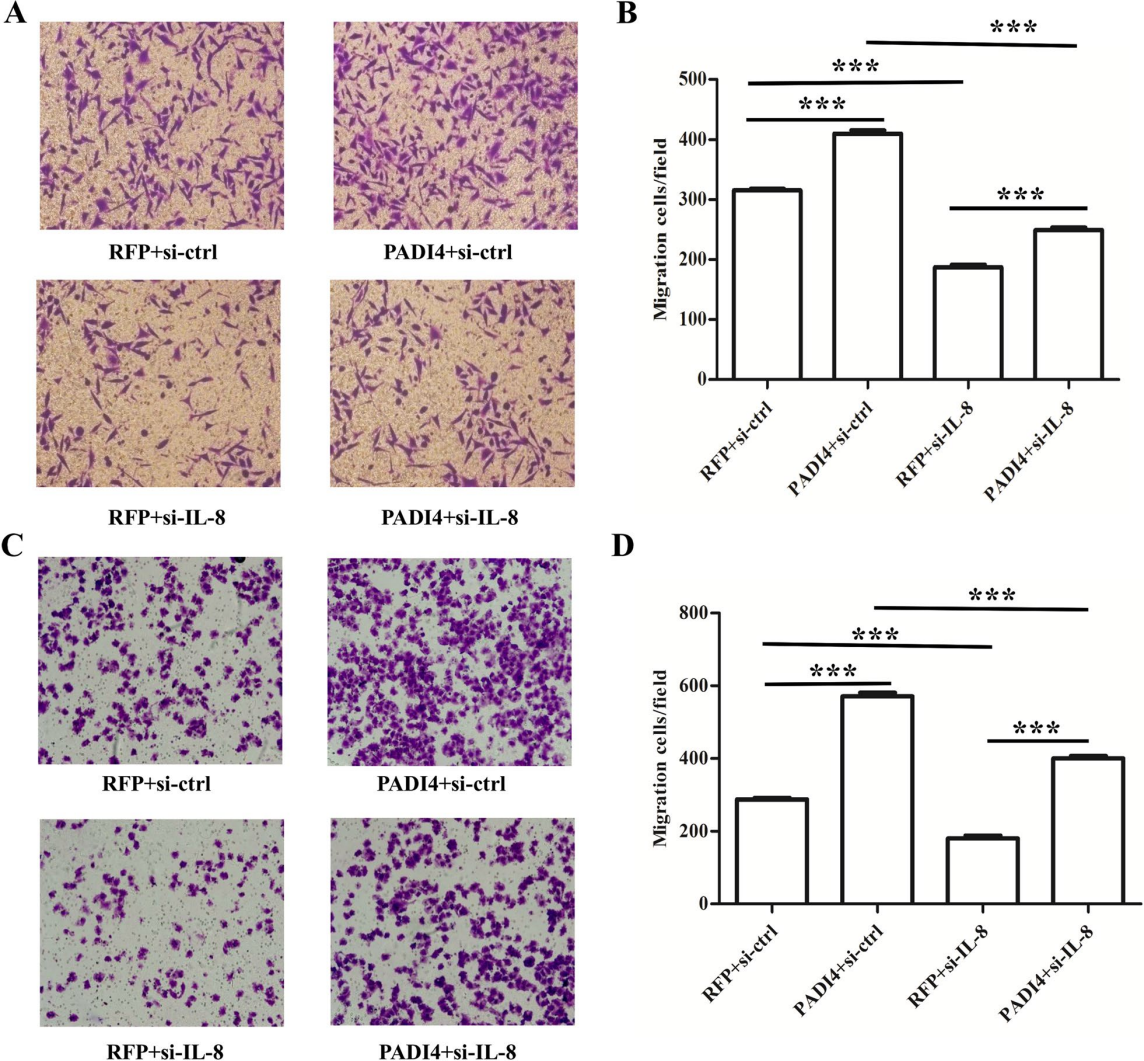
IL-8 is required for PADI4-mediated migration. Cell migration was determined by Transwell assay in PADI4-overexpressing MGC-803 cells (**A**, **B**). Cell migration was determined by Transwell assay in PADI4-overexpressing SGC-7901 cells (**C**, **D**). Original magnification: 400×

We found that silencing IL-8 significantly decreased the levels of IL-8 and vimentin in GC cells compared with nonsilencing siRNA GC cells (*P* < 0.05; Fig. 6). Moreover, Transwell results showed that silencing IL-8 expression significantly inhibited the increased metastatic capacity in PADI4-overexpressing GC cells (*P* < 0.05; Fig. 7).

## Discussion

GC has the characteristics of high incidence rate, high mortality rate and poor prognosis [22]. The reason for the high mortality is that most patients with GC have an advanced stage or metastasis, thus losing the best opportunity for treatment [23]. Although the surgical success rate and postoperative survival rate for tumours have been significantly improved at present, metastatic cancer patients still have higher drug resistance, poor treatment effect and low survival rate [24]. Therefore, the treatment of gastric cancer is still a high challenge.

PADI4 can generate of citrullinated proteins through convert peptidylarginine to peptidylcitrulline in the presence of Ca^2+^ [12], leading to the change of protein conformation and biochemical activity [25]. In rheumatoid arthritis (RA), PADI4 increases thrombin activity by citrullinating antithrombin, and thereby affects the vascular endothelial cells proliferation and RA synovial tissue inflammation [26, 27]. PADI4 also plays an important role in tumorigenesis and pathogenesis in lung cancer, colorectal cancer, oesophageal cancer and ovarian carcinoma [7, 13, 28, 29].

In this study, we found the expression of PADI4 was elevated in GC tissues. PADI4 play importance roles in regulating the cell proliferation, migration, colony formation capabilities and apoptosis capability of different degrees of differentiated cells. PADI4 also can accelerates cell activity and tumour growth in tumour-bearing tissues in vivo. This is consistent with the role of PADI4 in other cancers. Therefore, PADI4 has an oncogenic role in GC and can be a prognostic indicator of GC.

PADI4 have been reported play an important role in the pathogenesis of lung cancer by mediating EMT [30, 31]. The characteristic of EMT is that it loses the adhesion ability of tumour epithelial cells and obtains the migration ability of mesenchymal cells, resulting in increased metastasis and drug resistance [5]. EMT plays a vital role in tumour metastasis. Our study found that the overexpression of PADI4 increased the migration ability of GC cells, increased the expression of the EMT markers vimentin and slug, but decreased E-cadherin expression. Those results suggest that PADI4 promotes cell migration in GC through EMT process. This is the first report to demonstrate a new role of PADI4 in EMT and cell metastasis in GC.

To clarify the molecular mechanism of PADI4 in GC progression, we used the Multi-Analyte ELISArray Kit to analyse the expression of cytokines in PADI4-transfected GC cells. In the Multi-Analyte ELISArray Kit results, IL-8 was upregulated in PADI4-overexpressing SGC-7901 and MGC80-3 cells. IL-8 is a chemokine that can stimulate the migration of leucocytes. In ovarian cancer, IL-8 can promote migration and EMT through Wnt/β-catenin signalling network [32]. Evidences shows that IL-8 involved in promoting migration of colon cancer, pancreatic cancer and other types of cancers [18, 33–35]. Meanwhile, the level of IL-8 has been proven to be an indicator of poor prognosis in GC [36]. However, the pathogenesis of IL-8 in GC remains unclear. In this study, we found that silencing IL-8 expression blocked the expression of the EMT marker vimentin in PADI4-overexpressing GC cells. Transwell assays also showed that IL-8 silencing significantly inhibited the increased migration ability of PADI4 overexpressing GC cells. PADI4 is markedly upregulated in GC tissues. Thus, this study indicates that PADI4 promotes GC cells migration and EMT progression by regulating the expression of IL-8. These findings reveal a key role of the PADI4/IL-8 signalling pathway in EMT and provide a possible molecular mechanism for gastric carcinogenesis.

## Conclusions

Based on our findings, this study demonstrated that PADI4 could promote cell proliferation, migration and colony formation capabilities and inhibit the apoptosis in GC cells. The migration of GC cells caused by PADI4 was reduced by interference with IL-8 expression. We suggest that PADI4 can accelerate GC migration mediated by IL-8. Therefore, PADI4 has an oncogenic role in GC and could be a new target for GC diagnosis and therapy.

## Data Availability

The data are available from the corresponding author upon request.

## Abbreviations

GC: Gastric cancer
EMT: Epithelial–mesenchymal transition
PADI4: Peptidyl arginine deiminase IV
IL-8: Interleukin 8
siRNA: Small interfering RNA
GAPDH: Glyceraldehyde-3-phosphate dehydrogenase
SDS-PAGE: Sodium dodecyl sulfate–polyacrylamide gel electrophoresis
PVDF: Polyvinylidene fluoride
TBS: Tris-buffered saline
HRP: Horseradish peroxidase
ECL: Enhanced chemiluminescence
CCK-8: Cell Counting Kit-8
FITC: Fluorescein isothiocyanate
IFN: Interferon
TNF: Tumour necrosis factor
GM-CSF: Granulocyte–macrophage colony-stimulating factor
SEs: Standard errors
